# Mechanisms for mutual support in motor interactions

**DOI:** 10.1038/s41598-021-82138-y

**Published:** 2021-02-04

**Authors:** Lucia Maria Sacheli, Margherita Adelaide Musco, Elisa Zazzera, Eraldo Paulesu

**Affiliations:** 1 Department of Psychology and Milan Center for Neuroscience (NeuroMi), University of Milano-Bicocca, Piazza dell’Ateneo Nuovo 1, 20126 Milano, Italy; 2 IRCCS Istituto Ortopedico Galeazzi, Milan, Italy

**Keywords:** Psychology, Human behaviour

## Abstract

What is the key to successful interaction? Is it sufficient to represent a common goal, or does the way our partner achieves that goal count as well? How do we react when our partner misbehaves? We used a turn-taking music-like task requiring participants to play sequences of notes together with a partner, and we investigated how people adapt to a partner’s error that violates their expectations. Errors consisted of either playing a wrong note of a sequence that the agents were playing together (thus preventing the achievement of the joint goal) or playing the expected note with an unexpected action. In both cases, we found post-error slowing and inaccuracy suggesting the participants’ implicit tendency to correct the partner’s error and produce the action that the partner should have done. We argue that these “joint” monitoring processes depend on the motor predictions made within a (dyadic) motor plan and may represent a basic mechanism for mutual support in motor interactions.

## Introduction

Acting together with others requires prospective planning. The continuous updating of expectations regarding the partner's next step allows for efficient mutual adaptation^1,2^: it may make the difference between losing or scoring a match point during a team game. The monitoring of others’ actions also permits error detection, that is, the ability to identify a mismatch^3,4^ between the expected and actual responses provided by the partner. Investigating the processes underlying the detection of a partner’s error may enable exploring how the partner’s actions are represented and what expectations they trigger during a joint action, when we coordinate with a partner to achieve a joint goal^5^. This issue becomes crucial when aiming at modeling smooth interactions, e.g., with artificial agents, where the implementation of representational and monitoring mechanisms plays a significant role^6,7^.

**Figure 1:**
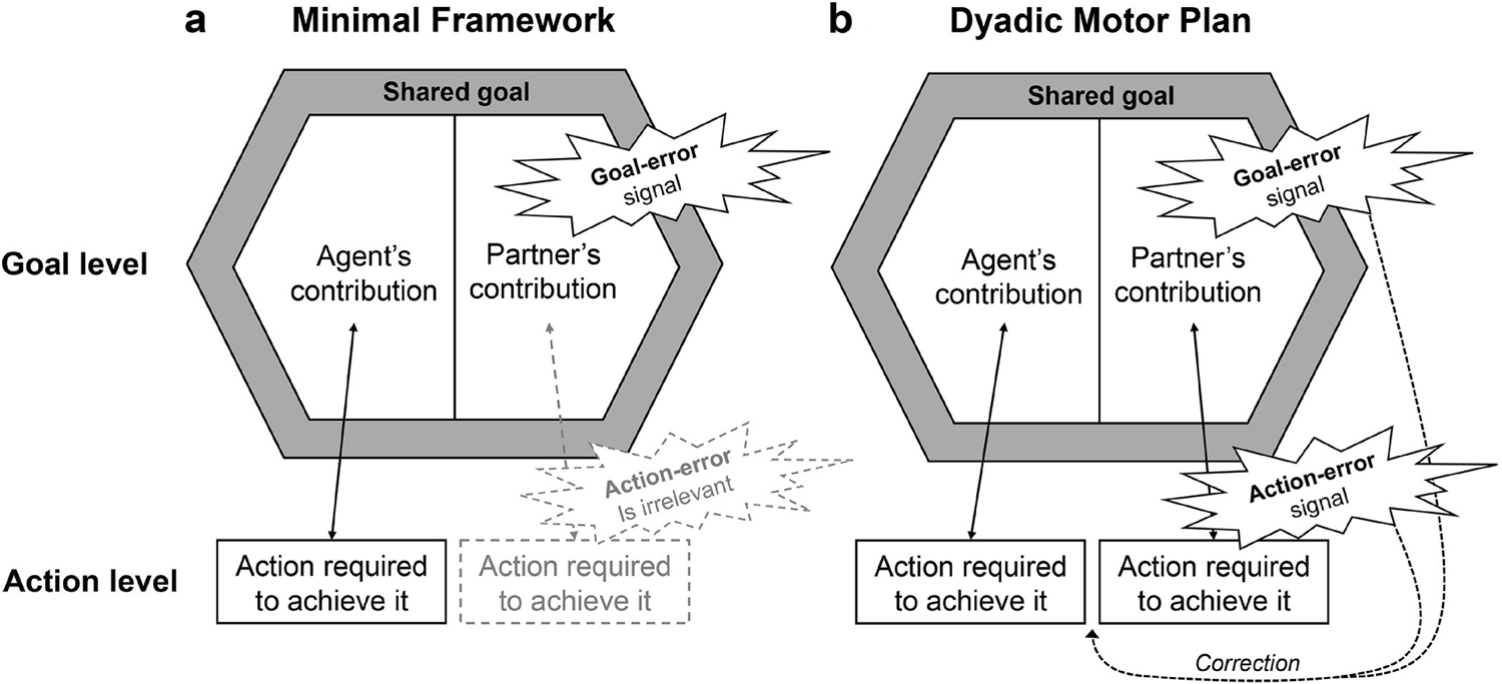
The figure illustrates the two alternative scenarios describing what motor representations might guide the monitoring of a partner’s error during a joint action, based on (**a**) a Minimal Framework for joint action, or (**b**) a Dyadic Motor Plan. Behaviorally, each time an error signal is present in the system, an observation-induced post-error slowing (oPES) effect is expected to emerge. According to the Minimal Framework, the oPES effect is only expected after errors that prevent the goal achievement; on the contrary, the Dyadic Motor Plan hypothesis suggests that Action-Errors could be also detected and trigger the tendency to correct the mistake (dotted lines on the right bottom of the figure).

In this regard, at least two alternative hypotheses can be put forward (Fig. 1). On the one hand, one might expect a negligible effect of expectations regarding how an overarching joint goal is achieved, provided that it is accomplished. Hence, a joint goal may be represented in rather abstract, non-motoric terms, without specific predictions on what actions the partner will perform to provide his/her contribution. For instance, during a volleyball match, players may focus on the ball trajectory by checking that it follows the playbook and disregarding what specific team-mates’ actions it requires. According to this minimal framework hypothesis for joint action (Fig. 1a), only the joint goal and one's contribution to achieving it are considered (see the most minimal form of joint representation as proposed by Vesper and co-authors^8^). In principle, the minimal framework is exceptionally efficient: it avoids the interference derived from the continuous error signals that would be generated each time the partner did not entirely match the agent’s expectations.

On the other hand, research on motor control has long established that motor planning hierarchies are strictly interconnected by predictive processing^9–11^. Thus, an overarching goal might trigger motor expectations of what specific actions are required from each agent^12–14^. Accordingly, having a playbook in mind would trigger specific expectations of the team-mates’ upcoming behavior. This interpretation would be in line with our and others’ suggestion that one’s and the partner’s actions are integrated within a dyadic motor plan (DMP^15,16^, Fig. 1b), resembling what happens for left- and right-hand movements during bimanual coordination^17–20^. The agents would then apply similar sensorimotor control processes to both their own and their partner’s actions^12,13^.

In the present study, we challenged the DMP and the Minimal Framework hypotheses by studying how people adapt to a partner’s error that violates either action- or goal-related expectations regarding the partner’s behavior. We reasoned that, according to the DMP hypothesis, both types of error would generate behavioral adaptations in the agent, while only a goal-related error should be detected according to the Minimal Framework hypothesis (Fig. 1).

Previous studies^21–24^ in the error detection domain investigated the so-called “observation-related post-error slowing” (oPES^25^, see also Ref.^26,27^) effect, that is, a slowdown in reaction times recorded after observing an error made by another person. This effect resembles what occurs after the self-generated errors^28,29^. The oPES effect suggests that not only similar neural structures are involved in both one’s and others’ error monitoring^30–34^, but also the post-error behavioral adaptations can be comparable. Crucially, however, this previous behavioral evidence was based on speeded choice reaction-time tasks like the flanker^21^ or go/no-go^22,26^ tasks, in which the observed error corresponded to a wrong response that prevented goal achievement. Hence, the experimental designs did not permit us to evaluate whether the onlooker’s representation is about the goal to be achieved, its causing action, or the combination of the two. As such, they do not allow one to decide on whether we collaborate with others by following a minimal framework scheme focused on goals (Fig. 1a), or by generating dyadic motor plans whereby it also counts what specific partner’s actions lead to the goal achievement (Fig. 1b).

Moreover, previous studies neglected a key feature of the behavioral adaptations following self-generated errors: their association with a prepotent tendency to correct mistakes^35,36^. It has been found that post-error behavioral effects disappear when participants are allowed to correct their mistake^35^ or perform a post-error trial requiring a response that matches the one that they should have made on the previous trial (i.e., which corresponds to a correction^36^). One might expect a similar pattern for observed errors in joint action: the oPES might be reduced when our actions match with the ones that our partner should have done, that is, when our response matches with a hypothetical “correction” of our partner’s error. This would indicate that, in line with the DMP hypothesis, the action monitoring mechanisms applied to our partner’s actions include a prepotent correction tendency (Fig. 1b, dotted lines).

In the present study, we addressed these issues and compared the Minimal Framework for joint action with the DMP account by investigating the oPES effect and spontaneous tendencies to correct a partner’s mistake. During a Joint Action task, we asked the participants to play short “melodies” (pre-learned four-note sequences) together with a virtual partner by playing with the computer mouse one note each in turn-taking (modified from Ref.^15^). At each trial, the participants were presented with a color cue indicating which four-note sequence they had to play together with the partner, thus creating an expectation on what notes the partner would play (see Suppl. Videos 1–2). The manipulation (introduced in 50% of the trials) of the partner’s association between the response buttons and the produced notes allowed us to create two kinds of violations of expectation. The manipulation led to partner’s “errors” that either (1) violated the expected musical sequence while yet respecting the sequence of button presses (Goal-Error), or (2) violated the sequence of expected button presses while yet maintaining the correct musical sequence (Action-Error). For instance, if a target musical sequence required the partner and the participant to play two C notes with the index finger and two G notes with the middle finger in turn-taking (C_index_, C_index_, G_middle_, G_middle_ sequence), in the Goal-Error condition, the partner would play an unexpected note with the correct finger (G_index_, C_index_, C_middle_, G_middle_ sequence), and in the Action-Error condition, the partner would play the correct note with an unexpected finger (C_middle_, C_index_, G_index_, G_middle_ sequence).

We aimed to test whether these two different error conditions induce an oPES to the same extent. We hypothesized that, although only violations in the musical sequences (Goal-Errors) really prevent the achievement of the joint goal and should then induce an oPES according to the Minimal Framework hypothesis (Fig. 1a), Action-Errors might have the same effect according to the DMP account (Fig. 1b). Knowing the joint goal (the melody) might indeed trigger expectations regarding what actions the partner should perform to provide his contribution (his note), then generating a mismatch, and therefore an error signal and an oPES, when such expectations are violated.

**Figure 2:**
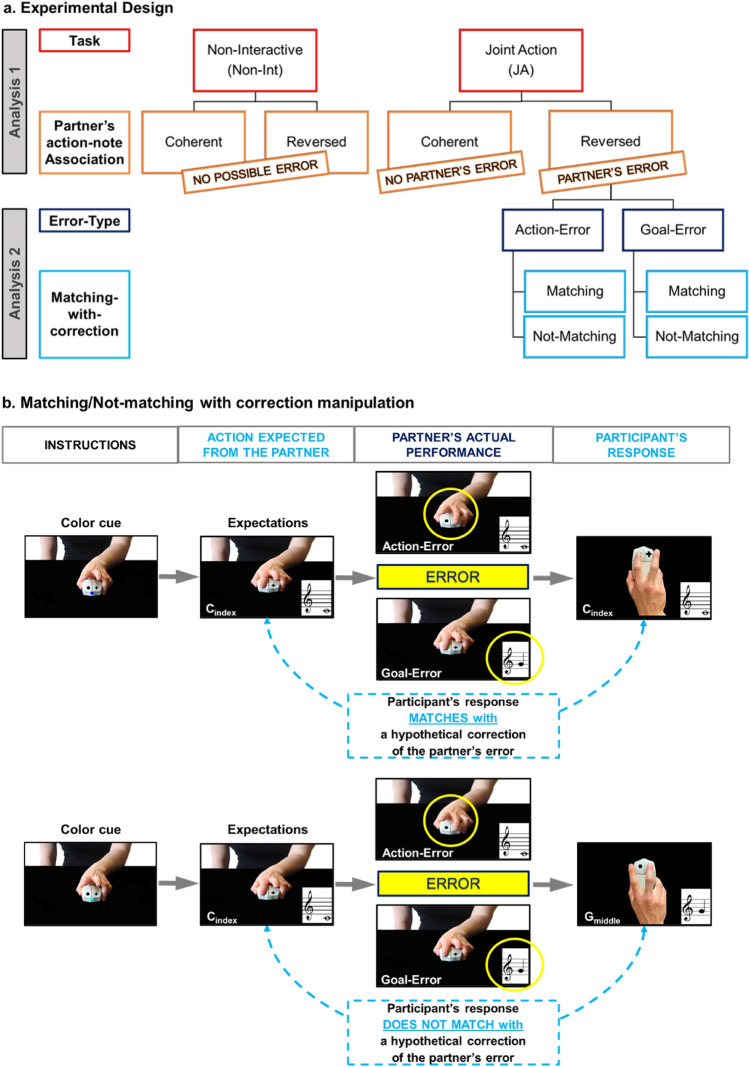
A schematic representation of the whole experimental design and the rationale of the Matching-with-correction manipulation. (**a**) The experiment included two tasks, the Joint Action (JA) and the Non-Interactive task. In both tasks, the association between the partner’s keypress action and the ensuing note was reversed (as compared to the partner’s one) in the 50% of the trials (Reversed-Association condition). Only in the JA task, the participants developed expectations about the actions and notes that the partner had to perform at each trial, because the color cue signaled what musical sequence the partner and the participant had to play *together*: thus, only in the JA task, the Reversed Association trials determined a violation of such expectations (“partner’s error trials”), while in Coherent Association trials no violation occured (“no partner’s error trials”). The JA Reversed Association trials included two possible Error-types made by the partner: Goal-Errors (i.e., the partner produces an unexpected note via an expected keypress action) and Action-Errors (i.e., the partner produces an expected note via an unexpected keypress action). (**b**) Independently of the Error-type, the response required from the participant in the JA Reversed Association trials could either match or not with the response that the partner was expected to (but did not) do: in principle, the participant’s responses that match with the expected (and not performed) partner’s responses correspond to what would be a correction of the partner’s error. See the main text for a detailed comment on the examples reported in the figure. In the analyses, we first investigated whether the reversal of the partner’s action-note association had a different impact on performance depending on the Task interactivity (*Analysis1*, upper part of the schema of the experimental design); then, we focused on the JA task and investigated the effect of Error-type and Correction Tendencies in Joint Action (*Analysis 2*, lower part of the schema of the experimental design).

Importantly, according to the DMP hypothesis, the oPES should also be reduced in trials where the participant’s response matches with a hypothetical “correction” of the partner’s error, in line with what happens for self-generated errors^35,36^. To test this latter point, we introduced a second manipulation: we coded all trials following a partner’s error depending on whether the participant’s action matched or not the hypothetical correction of the partner’s mistake. Importantly, this manipulation was independent of the Error-type, as it took place in both Action-Error and Goal-Error trials. In other words, independently of the type of error the partner performed, we coded the trials depending on whether the response requested from the participant matched with a hypothetical correction of the preceding partner’s mistake. Matching and Not-Matching trials were equally distributed: JA Reversed Association trials included 25% of Action-Error–Matching-with-correction trials, 25% of Action-Error–Not-Matching-with-correction trials, 25% of Goal-Error–Matching-with-correction trials, 25% of Goal-Error–Not-Matching-with-correction trials. See Fig. 2a for a scheme of the whole experimental design and Fig. 2b for an illustration of the Matching/Non-Matching-with-correction factor. As shown in Fig. 2b, the Matching-/Not-Matching-with-correction factor depends on what the partner’s expected performance is (see expectations in the figure), rather than on what the participants actually observe or hear at each trial. In the Matching-with-correction example (Fig. 2b, top raw), the response required from the participant (a C note, achieved by pressing the left key with the index finger) “matches” with the action that the participant should perform if he/she wanted to correct the partner’s error, independently of whether the error concerned the keypress action (Action-Error) or the note (Goal-Error). As the correction of the error is nothing but the partner’s expected performance, we can also say that in the Matching-with-correction trials the participant behavior matches with the one that the partner was expected to (but did not) perform. In the Not-Matching-with-correction example (Fig. 2b, bottom raw), the partner is expected to (but does not) play a C-note with the index finger, while the participant has to play a G-note with the middle finger. Thus, the hypothetical correction of the partner’s error (i.e., playing a C-note with the index finger) does not match with the response required from the participant.

For a better understanding of the manipulations, please refer to the Methods sections for a detailed description of the trial timeline and to the Supplementary Videos 3–6.

Participants also performed a control task (Non-Interactive task) requiring them to play a pair of notes independently of what notes the partner was playing, while still respecting the turn-taking format of the task. Here, the instructions did not specify what the partner would do, and the partner could not make proper "errors" because no expectation was present. However, this control condition allowed us to test whether, in a non-interactive context, the participant would note the reversal of action-note association in the partner or completely ignore it and only show an effect of spatial/movement compatibility, in line with our previous results^15^. This would confirm that pure action-effect associative learning cannot explain the interfering effect of the reversal of the partner’s action-note association that we expected to find in the Joint Action task.

## Results

The analyses were performed in two steps (see also Fig. 2a). First, we ran a preliminary analysis (Analysis 1, see “Methods”) on the Effect of Task. Analysis 1 aimed to test whether the effect of the reversal of action-note associations in the partner could be detected only in the Joint Action (JA) and not in the Non-Interactive (Non-Int) condition, and that this was independent of the perceptual congruence between the actual partner’ and participant’s responses at each trial. These results would replicate with the present experimental set-up our previous findings^15^ and indicate that the participants apply behavioral adaptations to the reversal of the partner’s action-note association only in a Joint Action (JA) and not in a Non-Interactive (Non-Int) task.

Second, we focused on the data collected during the JA task only, and we tested the Effect of Error-type and Correction Tendencies in Joint Action (Analysis 2, see “Methods”) by comparing the participants’ performance in trials where the partner made no error (JA Coherent Association trials) with the one collected in trials following a partner’s error of different types (JA-Reversed Association trials, see Fig. 2a on the bottom right side). We tested whether the observation-induced post-error slowing (oPES) that we expected to find in Analysis1 in JA Reversed Association trials was equal in trials including a Goal- versus Action-Error; we also tested whether this expected oPES effect could be interpreted as the result of the tendency to correct the partner’s mistakes, paralleling what happens for self-generated errors^35,36^.

### Analysis 1: preliminary analysis on the effect of task (JA vs. Non-Int)

For the sake of clarity, we report in Table 1 the group mean accuracy (ACC) and reaction times (RTs) raw data: here, we calculated the individual mean ACC and RTs per each experimental condition, excluding from the calculation of RTs the outlier values that fell 2.5 SDs above or below each individual's mean for each experimental condition calculated in accurate trials only.

**Table 1:** Group mean accuracy and reaction times values in analysis 1; data are classified depending on the Task (JA vs. Non-Int), Association (Coherent vs. Reversed), and Congruency in action and space (Congruent vs. Incongruent).

	JA-Coh-C	JA-Coh-In	JA-Rev-C	JA-Rev-In	NI-Coh-C	NI-Coh-In	NI-Rev-C	NI-Rev-In
**Accuracy**								
Mean	0.97	0.97	0.95	0.94	0.99	0.99	0.99	0.98
Range	1.00–0.88	1.00–0.89	1.00–0.83	1.00–0.85	1.00–0.96	1.00–0.93	1.00–0.96	1.00–0.93
**Reaction times (ms)**								
Mean	554.87	575.05	640.48	640.28	391.99	397.81	379.41	401.08
SD	188.55	164.06	186.29	198.06	153.37	133.21	137.44	139.50

*JA* joint action task, *NI* non-interactive task, *Coh* coherent association, *Rev* reversed association, *C* congruent, *In* incongruent.

#### Accuracy (ACC)

**Figure 3:**
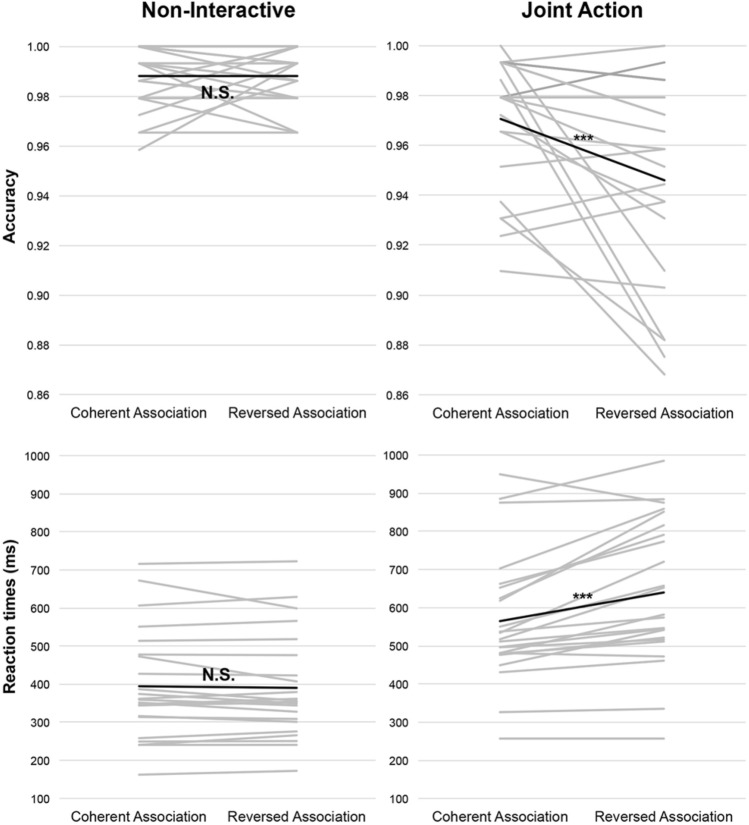
The figure illustrates the results that emerged from Analysis 1 on the Accuracy and Reaction Times data. The grey lines plot the individual effects of Association and the black lines indicate the group means.

The best fitting model included Task, Association, Congruency, and their interactions, as fixed effects (Suppl. Table S1). The results showed a significant main effect of Task (Wald Z = 4.06, *p* < 0.001) and Association (Wald Z = − 2.41, *p* = 0.016). These main effects indicated that the participants were more accurate in the Non-Int than the JA task (Non-Int adjusted (adj) mean 0.990, SE 0.18, vs. JA adj mean 0.966, SE 0.15), and in trials in which the partner's action-note association was Coherent than Reversed (Coherent Association adj mean 0.984, SE 0.17, vs. Reversed Association adj mean 0.979, SE 0.17). Importantly, the results also showed the Task × Association significant interaction (Wald Z = 2.05, *p* = 0.04) indicating that, as expected, the effect of Association was significant only in the JA task (Coherent Association adj mean 0.975, SE 0.17, vs. Reversed Association adj mean 0.954, SE 0.16, *p*_corr_ < 0.001) and not in the Non-Int task (Coherent Association adj mean 0.990, SE 0.21, vs. Reversed Association adj mean 0.991, SE 0.22, *p*_corr_ > 0.9). See Fig. 3. All other effects were not significant (all *p*s > 0.09).

For the sake of clarity, and given the high significance of the main effect of the Task in the omnibus analysis reported above, we also ran two separate analyses per each Task.

##### Non-Int task data

The best fitting model only included Congruency (Congruent vs. Incongruent action and space) as fixed effect (Suppl. Table S1). The significant effect of Congruency (Wald Z = − 2.19, *p* = 0.028) indicated that the participants were more accurate in the Congruent than the Incongruent condition (adj mean 0.993, SE 0.26, vs. 0.988, SE 0.23).

##### JA task data

The best fitting model only included Association (Coherent vs. Reversed) as fixed effect (Suppl. Table S1). The significant main effect of Association (Wald Z = − 5.12, *p* < 0.001) indicated that the participants were more accurate in the Coherent than the Reversed Association condition (adj mean 0.976, SE 0.19, vs. 0.956, SE 0.18).

#### Reaction times (RTs)

The best fitting model included Task, Association, and their interactions, as fixed effects (Suppl. Table S2). The results showed a significant main effect of Task (F(1, 12432) = 1125.71, *p* < 0.001) and Association (F(1, 12432) = 30.75, *p* < 0.001), indicating that the participants were faster in the Non-Int than the JA task (adj mean 392.42 ms, SE 22.42 ms, vs. 598.02 ms, SE 22.44 ms), and in trials in which the partner's action-note association was Coherent than Reversed (adj mean 478.23 ms, SE 22.42 ms, vs. 512.21 ms, SE 22.43 ms). The results also showed the significant Task x Association interaction (F(1, 12432) = 38.81, *p* < 0.001) showing that, as expected, the effect of Association was significant only in the JA task (Coherent Association adj mean 561.94 ms, SE 22.85 ms, vs. Reversed Association adj mean 634.10 ms, SE 22.87 ms, *p*_corr_ < 0.001) and not in the Non-Int task (Coherent Association adj mean 394.52 s, SE 22.83, vs. Reversed Association adj mean 390.31 s, SE 22.83, *p*_corr_ > 0.9). See Fig. 3.

For the sake of clarity, and given the high significance of the main effect of the Task in the omnibus analysis reported above, we also ran two separate analyses per each Task.

##### Non-Int task data

The best fitting model only included Congruency (Congruent vs. Incongruent action and space) as fixed effect (Suppl. Table S2). The significant main effect of Congruency (F(1,6306) = 8.05, *p* = 0.004) indicated that the participants were faster in the Congruent than the Incongruent condition (Congruent trials adj mean 385.46 ms, SE 29.14 ms, vs. Incongruent trials adj mean 399.05 ms, SE 29.14 ms).

##### JA task data

The best fitting model only included Association (Coherent vs. Reversed) as fixed effect (Suppl. Table S2). The significant main effect of Association (F(1, 6104.1) = 47.48, *p* < 0.001) indicated that the participants were faster in the Coherent than the Reversed Association trials (Coherent Association adj mean 563.43 ms, SE 36.44 ms, vs. Reversed Association adj mean 637.06 ms, SE 36.46 ms).

### Analysis 2: the effect of Error-type and correction tendencies in joint action

#### Analysis of the Error-type factor

First, we considered as fixed effect only the Error-type (3 levels: trials following a partner’s action with no error vs. Action-Error vs. Goal-Error; see Suppl. Videos 3–6). With regard to Accuracy, the results showed that the participants were more accurate in the trials following a partner’s correct action (adj mean 0.976, SE 0.19) than in the trials following a partner’s error (both *p*s < 0.001), and that the two error conditions did not differ between each other (Action-Error adj mean 0.959, SE 0.19, Goal-Error adj mean 0.953, SE 0.19; *p* > 0.9). With regard to the Reaction Times, the results showed that the participants were faster in trials following a partner’s correct action (adj mean 563.43 ms, SE 36.43 ms) than in the trials following a partner’s error (both *p*s < 0.001), and that the two error conditions did not differ between each other (Action-Error adj mean 646.52 ms, SE 37.23 ms; Goal-Error adj mean 627.54, SE 37.24 ms; *p* = 0.636).

**Figure 4:**
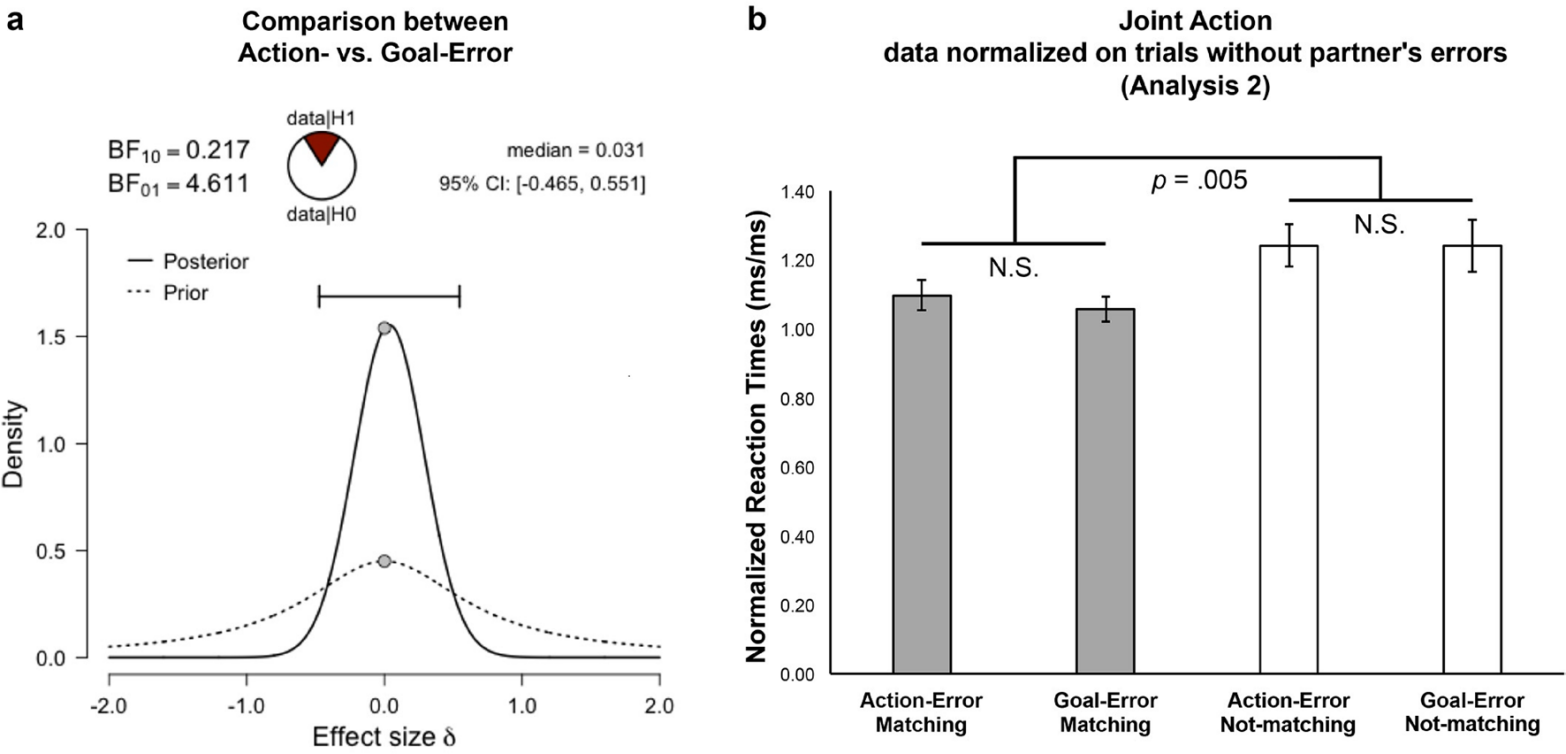
The figure illustrates the results that emerged from Analysis 2. On the left (**a**), the results of the Bayesian paired-sample t-tests that was run on the comparison between the Reaction Times recorded after the partner’s Action- versus Goal-Errors. On the right (**b**), the graph shows the group means of normalized Reaction Times: here, each experimental condition in which the participants observed a partner’s error was normalized on (divided by) the performance recorded in the trials following a partner’s correct action.

Given the lack of a significant difference between the participants’ performance following a partner’s Action- and Goal-Error, we applied a Bayesian approach to assess the strength of evidence in favor of the null hypothesis. We compared with a Bayesian paired-sample t-test the individual mean ACC and RTs in the Action- versus Goal-Error conditions. The Bayesian Factor (BF10) was lower than 0.3 in both Accuracy (mean ACC Action-Error 0.953 ± 0.05; mean ACC Goal-Error 0.946 ± 0.06, BF10 = 0.246) and Reaction Times (mean RTs Action-Error 655.77 ± 217.67 ms; mean RTs Goal-Error 649.92 ± 228.17 ms, BF10 = 0.217). See Fig. 4a. These results show moderate evidence in favor of the null hypothesis, that is, the absence of difference in performance between trials following a partner’s Action- versus Goal-Error in the JA task.

#### Analysis of the Matching-with-correction factor

Second, we considered as fixed effect the matching with a hypothetical correction of the partner’s error (3 levels: trials following a partner’s action with no error vs. trials where the participant’s action was Matching-with-correction vs. Not-Matching-with-correction, see Suppl. Videos 3–6). We reasoned that, if the same monitoring mechanisms are applied to the self and the partner in joint action as suggested by the DMP hypothesis, one should expect to find an oPES effect that could be interpreted as the result of the participants’ tendency to correct the partner’s errors, paralleling what happens for self-generated errors^35,36^. Thus, the oPES should be reduced when the participant’s response matches the one that the partner was expected to perform right before (Matching condition), as if the participant attempted to correct the partner’s mistake (see Fig. 2b).

With regard to Accuracy, the results showed that the participants were more accurate in the trials following a partner’s correct action (adj mean 0.976, SE 0.19) than in the trials where the participant’s action did not match with a hypothetical correction of the partner’s error (Not-Matching-with-correction adj mean 0.940, SE 0.18, *p* < 0.001), but not when it matched such a hypothetical correction (Matching-with-correction adj mean 0.979, SE 0.20, *p* = 0.54). As a matter of fact, the participants were more accurate in the Matching- than the Not-Matching-with-correction trials (*p* < 0.001). With regard to the Reaction Times, the results showed that the participants were faster in the trials following a partner’s correct action (adj mean 563.44 ms, SE 36.47 ms) than in the trials following a partner’s error (Matching adj mean 605.23 ms, SE 37.23 ms, *p* = 0.004; Not-Matching, adj mean 670.56 ms, SE 37.31 ms, *p* < 0.001). RTs in the Matching- and Not-Matching-with-correct trials also differed from each other, as the participants were faster in the former (*p* < 0.001). These results suggest that participants make active use of their expectations regarding what the partner is supposed to do: these expectations are supported by the predictive mechanisms constituting the dyadic motor plan, and lead to behavioral adaptations in case of a violation (i.e., the oPES effect) that are compatible with a spontaneous tendency to correct it by performing the action that the partner should have done.

#### Analysis of a possible interaction effect between the Error-type and the Matching-with-correction factors

Finally, we tested whether, despite the absence of a significant difference between performance following a partner’s Action- versus Goal-Error, the Error-type might nevertheless modulate the effect of Matching. That is, we explored the possible presence of an interaction effect between the factors Matching-with-correction and Error-type. We normalized the participant’s performance in each condition following a partner’s error (i.e., Matching-with-correction in Action-Error, Matching-with-correction in Goal-Error, Not-Matching-with-correction in Action-Error, Not-Matching-with-correction in Goal-Error) by dividing it by the participant’s performance in trials following a partner’s correct action (see Methods). Then, we ran an ANOVA having Matching (2 levels: Matching- and Not-Matching-with-correction) and Error-type (2 levels: Action- and Goal-Error) as within-subject factors. This analysis was run on individual mean RTs values. To control for the presence of speed-accuracy trade-offs, we also calculated the same ratios on individuals’ mean ACC values and then ran an analogous ANOVA on Inverse Efficiency Scores (IES^37^). The group means per condition of normalized RTs, ACC and IESs are reported in Table 2.

**Table 2:** The group mean ACC, RTs and IESs values that entered the analysis to explore the possible presence of an interaction effect between Matching-with-correction and Error-type (last step of Analysis 2).

	Action-Error, Match	Action-Error, Mismatch	Goal-Error, Match	Goal-Error, Mismatch
**Normalized (error/correct) accuracy**				
Mean	0.99	0.97	1.00	0.95
SD	0.01	0.01	0.01	0.02
**Normalized (error/correct) reaction times**				
Mean	1.10	1.24	1.06	1.24
SD	0.04	0.06	0.04	0.08
**Normalized (error/correct) inverse efficiency scores**				
Mean	1.12	1.31	1.06	1.41
SD	0.06	0.08	0.03	0.16

Match = Matching-with-correction condition; Mismatch = Not-Matching-with-correction condition. Here, the participant’s performance in each condition following a partner’s error (i.e., Matching-with-correction in Action-Error, Matching-with-correction in Goal-Error, Not-Matching-with-correction in Action-Error, Not-Matching-with-correction in Goal-Error) was normalized by dividing it by the participant’s performance in trials following a partner’s correct action.

The results showed a significant effect of Matching in both RTs (F(1,22) = 9.71, *p* = 0.005, _p_η^2^ = 0.31) and IESs (F(1,22) = 10.87, *p* = 0.003, _p_η^2^ = 0.33), and the absence of a significant effect of Error-type and Matching x Error-type interaction (RTs, all *p*s > 0.56; IESs, all *p*s > 0.28). The Bayesian paired-sample t-test that was run on the Error-type comparisons (Action- vs. Goal-Error) separately for the Matching- and Not-Matching-with-correction trials also replicated the results reported above. The comparison showed evidence in favor of the null hypothesis in both the Matching- (RTs BF10 = 0.288; IES BF10 = 0.312) and the Not-Matching-with-correction trials (RTs BF10 = 0.219; IES BF10 = 0.250). See Fig. 4.

### Replication experiment

In the Supplementary Information we report the behavioral results of a replication experiment (N = 24) in which an identical experimental paradigm was adapted to the MRI environment. As the reader shall see, the results fully replicated those reported in the main text of this manuscript.

## Discussion

The present study aimed to explore what expectations guide motor planning in a joint action by investigating the participants’ ability to detect a partner’s error and its impact on performance. The rationale behind this approach is that the agents would notice a partner’s error and show consequent behavioral adaptations only if they had specific expectations regarding the partner’s behavior. We compared two alternative hypotheses on what guides motor planning and monitoring in joint action: a Minimal Framework or a Dyadic Motor Plan (DMP).

A Minimal Framework for joint action would have been satisfied by the agent considering just the shared goal and his/her contribution to it (in our paradigm, the required musical notes), regardless of any motor coding of how this contribution is provided by the partner (in our paradigm, what button presses generate the notes). On the contrary, our DMP hypothesis^15^ suggests that the knowledge of the shared goal should trigger motor expectations regarding the partner’s actions as if they were part of the agent’s plan. Thus, the agent would detect not only the Goal-Errors that prevent the goal achievement (the correct melody), but also the Action-Errors that lead to the goal achievement via unexpected actions. Moreover, the DMP hypothesis predicts that this error detection should trigger the same monitoring and remedial processes that take place after self-generated errors^35,36^, which suggest an implicit tendency to correct the violation of expectations by performing the action that should have been done.

It is worth noticing that, in our task, the behavior predicted by the Minimal Framework would be more efficient, as it would ensure saving attentional resources by disregarding the partner's specific actions to only focus on goal achievement. In principle, the participants could have even disregarded the partner’s performance entirely, because they knew (through the color cue) what musical sequence they had to play before observing any partner’s action.

The results were yet in favor of the DMP hypothesis. Indeed, they showed strong evidence of the presence of participants’ behavioral adaptations to the partner’s errors (i.e., the observation-induced post-error slowing effect, oPES) which were independent of the type of error observed, i.e., regardless of whether it was an Action- or a Goal-Error. We interpret these behavioral adaptations as the result of the participants’ tendency to correct the partner’s erroneous/unexpected behavior, following the same rationale that previous studies have applied to investigate similar phenomena in self-generated errors^36^. That is, in the trials following a partner’s error, the participants' performance changed depending on whether they had to perform an action that corresponded to a hypothetical correction of the partner's mistake (Matching- vs. Not-Matching-with-correction trials, see Fig. 2b and Suppl. Videos 3–6). The oPES was maximal in the Not-Matching-with-correction trials, where participants were also less accurate. This indicates that, despite an overall impact of a violation of expectations^38^, such impact was reduced when the participants were required to perform an action and note that matched the one that the partner should have done (Matching-with-correction trials). The stronger performance decay measured in the Not-Matching trials could then be interpreted as evidence of the participants’ need to inhibit the tendency to correct the partner’s action in order to perform a different response. This correction tendency is most likely not overtly intentional—this is why we label it as implicit—and it may purely depend on the predictive cascades that take place in motor control, which here apply to the partner’s actions as well (see Ref.^12^).

It is worth noticing that the behavioral difference in performance between the Matching- and Not-Matching-with-correction trials cannot be explained by perceptual congruence, that is, the congruence between the partner’s and the following participant’s actions, for two main reasons. First, Analysis 1 showed that Congruency did not play any role in the Joint Action task. This excludes the possibility that what we interpret as an implicit tendency to correct the partner’s errors is merely due to low-level priming effects associated with the physical congruence between the items in the sequence (e.g., C_index_ C_index_ rather than G_middle_ C_index_): if this was the case, we would have found an effect of Congruency in the Coherent-Association trials in JA, but we did not. Second, the Matching-with-correction trials imply actions (index- vs. middle-finger button presses) that are physically congruent with the partner’s ones in the Goal-Error trials (e.g., target sequence: C_index_ C_index_ G_middle_ G_middle_; Goal-Error sequence: G_index_ C_index_ C_middle_ G_middle_), while they are physically incongruent in the Action-Error trials (e.g. target sequence: C_index_ C_index_ G_middle_ G_middle_; Action-Error sequence: C_middle_ C_index_ G_index_ G_middle_). See Table 3. Since the facilitation observed in the Matching- as compared to the Not-Matching-with-correction trials was equally present following an Action- and a Goal-Error made by the partner, physical congruence of actions cannot account for it. Instead of being guided by what they actually see, the participants' behavior depends on their expectations of the partner’s action, and it shows an adaptation when such expectations are violated.

**Table 3:**
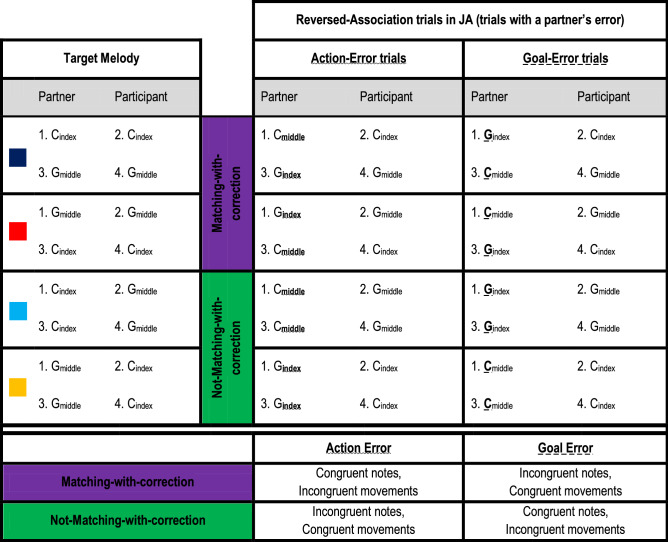
The Table illustrates, per each melody, what a sequence with Action- and Goal-Errors looked like.

Each melody included 4 subsequent notes: the partner always played the first and third ones, while the participant played the second and fourth ones. Per each melody, the first and second notes are reported in the top row of the cell (first half of the melody, which constitutes a trial), and the third and fourth ones in the bottom raw (second half of the melody, which constitutes another trial). If a partner’s error occurred in the melody, it was present both when the partner played the first and the third notes. The association between each color cue (shown on the left, next to each melody) and the specific melody was counterbalanced between participants. Importantly, as shown in the summary table below, the Matching-/Not-Matching with correction factor was *not systematically associated to perceptually congruent or incongruent actions or notes*. Indeed, the Matching condition included trials with congruent notes and incongruent movements in the case of Action-Errors, and incongruent notes and congruent movements in Goal-Errors. On the contrary, the Not-Matching condition included trials with incongruent notes and congruent movements in Action-Errors, and congruent notes and incongruent movements in Goal-Errors. Thus, the main effect of Matching- versus Not-Matching, which was equally present in Action- and Goal-Error trials, cannot be accounted for by the physical congruence between the partner’ and the participant’s actions performed at each trial.

These results were entirely replicated by a second experiment (see Supplementary Results in the Supplementary Information).

Our results show that, during a joint action, knowing the joint goal triggers expectations regarding what specific actions the partner should perform to provide his/her contribution. When such expectations are violated, the resulting error signal triggers the motor representation of the action that would correct the mistake. Evidence of the lack of significant difference between Action- and Goal-Error trials strongly support the DMP hypothesis and the theoretical stand-points suggesting that the hierarchical levels of motor planning (from goals to movements) are intertwined in action observation and execution^9–11^, and particularly so in joint action, where shared goals guide action planning^12,13,39^. The present study demonstrates the genuinely motor nature of these “joint” (dyadic) planning processes, and it shows, for the first time, that the monitoring of a partner’s behavior includes spontaneous correction tendencies that resemble the ones that emerge for self-generated errors^35,36^.

The present findings also replicate our previous observations on the effect of task interactivity on action observation and its impact on the agent’s behavior, which we demonstrated in adults^15,16^ (see^40^ for an independent partial replication) and children^41^. In the Non-Interactive task, the participants had to play music in turn-taking with a partner who did not share a joint goal with them. Here, the partner cannot make any “error” as the color cue does not specify his actions, yet, in principle, participants might have noticed the reversal in his action-note associations. Instead, the results showed that this was not the case, and that in the Non-Interactive task the participants were only slower and less accurate when they produced an action that was physically incongruent with the one just produced by the partner, in line with previous studies on visuomotor interference effects^42,43^.

These findings highlight the predictive nature of action simulation^9,10^ and the social flexibility thereof^44–48^ (see also Ref.^49^). While physical congruence of actions interferes with motor execution when the partner’s action outcome is irrelevant and no expectation is present^15,16^, the outcome and the ensuing action-outcome associations are taken into account during interactions (see also Ref.^50^), possibly requiring additional neural resources. Our and others’ previous results suggest that this interactivity-dependent modulation of action observation might depend on a modulation of the brain activity in fronto-parietal areas^51–59^, see also Ref.^60^. Specifically, in the present paradigm, the left ventral premotor cortex might play a critical role^16^, because it is responsible for goal predictions for hand movements in humans^61,62^ and non-human primates^63,64^.

Importantly, the results on the effect of task interactivity (Analysis 1) also rule out that the participants’ behavior in the Joint Action task can be explained in terms of pure auditory-motor associative learning. Indeed, the effect of the reversal of the action-note association was not noticed in the Non-Interactive task, where pure associative learning could yet be at play.

The cognitive bases of the behavioral adaptations following self-generated errors are still a matter of debate^65^. Solving the debate is well beyond the scope of the present study, yet, we can highlight that in our task the participants showed an observation-induced post-error behavioral effect in both accuracy and reaction times, thus suggesting that it cannot be accounted for by an increased allocation of attention following the observation of an error as an infrequent/salient event^66^. First, the “errors” occurred 50% of the time (and were thus not infrequent, see also Ref.^67^); second, the longer reaction times were not coupled with improved accuracy, as it would have been expected in the case of increased attentional processing^66^. The results are instead coherent with an explanation in terms of proper planning of a remedial action^3,35,36^, as described above.

In our experimental task, the (involuntary) attempts to apply remedial actions to the partners’ erroneous/unexpected behavior imply a performance cost, and they may thus seem maladaptive. However, this mechanism may be useful in real-life interactions, where correcting a partner’s mistake might facilitate the achievement of the joint goal. The “correction tendencies” hence become adaptive processes in the light of the joint rather than the individual gain. These (cognitively costly) monitoring processes might be considered a rather basic mechanism for partners’ mutual support, as they allow remedying to the partner's slip-ups and scaffold his/her behavior; they might also support dyadic collaborative learning through trial and error joint optimization based on the reciprocal correction of one’s mistake.

Both joint actions and collective decision making^68,69^ are characterized by the agent’s investment in behavioral adaptations that imply costs in terms of cognitive and/or motor resources (e.g., deviation from maximal efficiency of movement trajectory^70–75^), which are nevertheless repaid by smoother coordination and higher co-efficiency (see Ref.^76,77^). Admittedly, we did not test in our task whether a joint payoff is what guides the agents’ joint motor planning, but our results suggest it might be a relevant avenue for future research in the field. For instance, the implementation of algorithms monitoring the agent’ and partner’s performance during human–robot interactions should include the implementation of costly remedial actions that yet maximize the joint action outcome, with a possible impact on the human commitment to the collaborative task^78^.

To conclude, our results indicate that joint action planning is not exclusively embedded in rather abstract mental representations in which the partner is the means to achieve an (otherwise unachievable) goal, as it might be the case for other species^79,80^. Instead, it is rooted in the agent’s sensorimotor representations, as the partner was “motorically embodied”^81^ by the agent. This social embodiment allows for those reciprocal collaborative adjustments that are critical for the interaction success.

Of course, we explored these effects within the time-window and constraints of an experimental tasks in which the motor range was reduced and controlled. Moreover, we cannot exclude that more “minimal” representations suffice in supporting interactions in some instances when one of the partners may not be able to represent the other’s or the joint goal (e.g., in infant-caregiver interactions)^8^. Thus, the conclusions taken from this task format may not generalize to all forms of motor interactions. Further studies are needed to clarify whether the Dyadic Motor Plan account still holds for interactions occurring at different time-scales, the neurophysiological processes underlying “dyadic” action monitoring, and the prosocial effects thereof (see Ref.^14,82–84^). However, the minimally prosocial tendency to scaffold our partner’s behavior and sacrifice efficiency for dyadic success may represent a building block for broader forms of collaboration.

## Methods

### Participants

24 participants took part in the study (11 males, age range 21–25, m = 22.25 ± 0.99). One subject was excluded from the analysis because she did not understand the task instructions in the Non-Interactive task (see below), thus showing extremely low accuracy (mean accuracy equal to 68% and just above chance). The remaining 23 participants (11 males, age range 21–25, m = 22.26 ± 1.00) were right-handed as confirmed by the Edinburgh Handedness Inventory (Oldfield, 1971; mean score 0.79 ± 0.16), reported normal or corrected-to-normal vision, absence of neurological or psychiatric disorders and were naive as to the purpose of the experiment. To determine the sample-size, we conducted in G*Power 3.1^85^ a power analysis based on the data of our previous study^15^ showing that, in a similar task, the impact on performance of a reversal of action-note associations in the partner is modulated by task interactivity. The analysis revealed that, with α = 0.05 and statistical power at 1–β = 0.95, we needed a sample size of N = 12 to replicate such an effect (paired sample t-test comparing the effect of Association between the interactive and non-interactive task). As we aimed to explore, within the interactive task, the presence of possible differences between different types of errors included in the reversed association condition, we doubled the sample-size. An identical sample-size was selected for the experiment described in the Main text and for the Replication experiment (see Supplementary Information).

The protocol was approved by the ethics committee of the University of Milano-Bicocca (Italy) and was carried out according to the ethical standards of the 1964 Declaration of Helsinki and later amendments. Participants gave their written informed consent to take part in the study in exchange for course credits and were debriefed as to the purpose of the study at the end of the experimental procedures. Professional musicians were not recruited.

### Stimuli and apparatus

Participants were comfortably seated in front of a rectangular (60 × 110 cm) table and watched a 1024 × 768 resolution LCD monitor placed on the table at a distance of ~ 60 cm from their eyes. A computer mouse was also placed on the table on the midline. Two black stickers were placed on the mouse buttons: a cross on the right one and a circle on the left one. Participants were instructed to press the right button with the middle finger and the left one with the index finger by using their right hand. During the experiment, touching the left or the right button would generate two different sounds, which were delivered to participants via headphones. The two sounds, of the same intensity (4 dB) and duration (100 ms), were either a C note (~ 261 Hz) or a G note (~ 392 Hz). A third, raspberry-like sound (duration = 100 ms) was also used as error signal.

The participants acted in response to visual stimuli, which differed in the two phases of the experiment. During the Learning phase (see below), small colored squares appeared on the computer screen.

**Figure 5:**
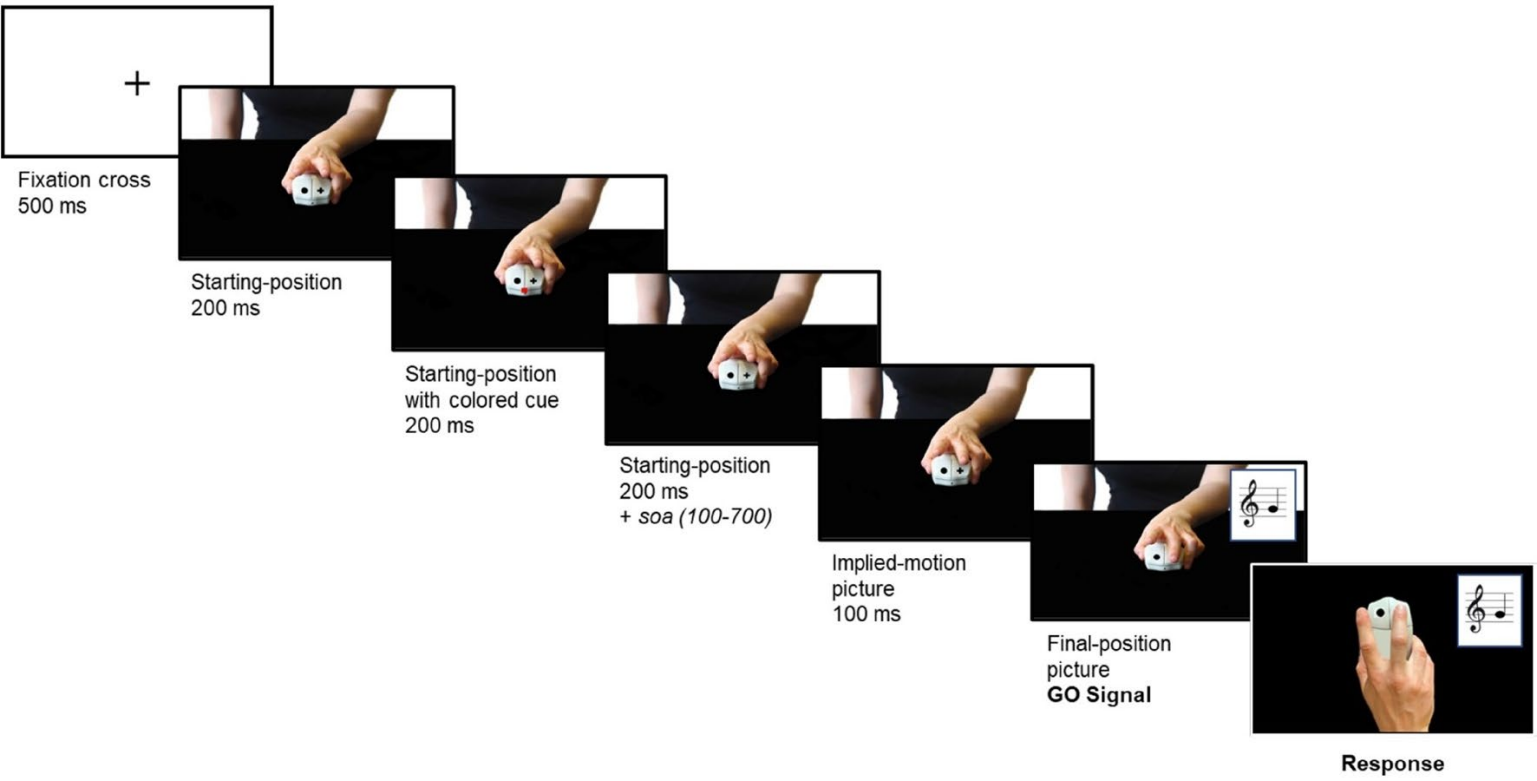
The figure illustrates the trial time-line, which was identical in the Joint Action and Non-Interactive task. At each trial, the participant saw a fixation cross followed by the image of the partner in the starting position; then, the color cue was presented for 200 ms at the center of the partner’s computer mouse; afterwards, the color cue disappeared while the starting-position image remained on the screen for additional 200 ms plus a variable stimulus onset asynchrony (soa); finally, the partner played his note, which constituted the participant’s go signal (see Suppl. Videos 1–6).

During the Test phase (see below), stimuli consisted of a sequence of pictures showing the left hand of a virtual partner holding a mouse similar to that of the subject. There were three different pictures (Fig. 5): (1) a starting-position picture (depicting the index and the middle finger lifted over the mouse), (2) an implied-motion posture picture (depicting the pressing-button actions at mid-flight), and (3) a final-position picture (depicting the end of the pressing-button action). The starting-position picture also included a version with a small colored square placed at the center of the partner’s computer mouse, which represented the color-cued instruction for the participant specifying the melody/pair of notes he/she had to perform (Fig. 5).

### Procedure

Stimuli presentation and randomization were controlled by E-Prime2 software (Psychology Software Tools Inc.).

#### Experimental tasks (Joint Action vs. Non-Interactive)^15,16^

There were separate sessions for the Joint Action (JA) and Non-Interactive (Non-Int) tasks, which were presented in counterbalanced order between the participants. During the two tasks, identical stimuli were presented, and participants were required to alternate with the partner in generating the notes using the computer mouse. The conditions only differed in the task instructions. In the JA condition, the color-cue (e.g., red, orange, blue, or light-blue) informed participants on which of four four-note melodies they had to play together with their partner, alternating in playing one note each: the participants thus played two of the four notes in turn-taking with the partner while keeping in mind the full melody. In the Non-Int condition, the color-cue (e.g., yellow, green, pink, or violet) was associated with one of four pairs of notes that participants had to play in two consecutive trials independently from what notes the partner was playing. For instance, for one participant the color-cue could specify: JA condition, red melody = C–C–G–G, orange melody = C–G–C–G, blue melody = G–G–C–C, light-blue melody = G–C–G–C; Non-Int condition, yellow pair = C–G; green pair = C–C; pink pair = G–C, violet pair = G–G (the association between colors and melodies/pairs of notes was counterbalanced between participants). Thus, all color-cues conveyed the same amount of information regarding what action the participant had to perform in two consecutive trials. For instance, in the example reported above both the red and the yellow cues informed the participants that they had to generate a C and then a G note; the color-cue in the JA condition yet also informed the participants on what the partner had to do, while in the Non-Int condition the partner’s action was not specified.

#### Experiment phases and conditions

Each JA/Non-Int task session was divided into two phases: Learning phase, and Test phase. Each JA/Non-Int Test phase was preceded by the corresponding Learning phase.

##### Learning phase

The Learning phase (about 20 min) was purely aimed to teach the participants the correct association between each color-cue and melody (in the JA task) or pair of notes (in the Non-Int task). Participants did not train the interactive task in this phase, as no partner was shown. As reported above, the structure of participants’ melodies and pairs of notes was as follows:

- Melodies (JA): (1) C–C–G–G, (2) C–G–C–G, (3) G–G–C–C, (4) G–C–G–C;
- Pairs of Notes (Non-Int): (1) C–G, (2) G–G, (3) G–C, (4) C–C.

Each Learning phase was divided into two parts. First, the participants heard each musical sequence (melody in the JA task, pair of notes in the Non-Int task) while being concurrently presented with the corresponding color cue, and they were asked to immediately reproduce it. Each musical sequence was consecutively presented until participants correctly reproduced it five consecutive times. Afterwards, participants were randomly presented the color cues and were required to produce the corresponding musical sequence. The color cue corresponding to each musical sequence was presented six times. Participants had to correctly produce each sequence at least five times (accuracy threshold equal to 80%) in order to move forward to the next part of the learning phase, otherwise they were asked to repeat the first part.

Then, participants performed a recognition task: they heard each musical sequence 10 times (random presentation) and had to identify the corresponding color cue. Participants had to correctly identify each musical sequence eight out of ten times (accuracy threshold equal to 80%) in order to move forward to the Test phase, otherwise they were asked to repeat the recognition task.

Only participants who successfully completed the Learning phase by repeating each part no more than three times could start the Test phase. No participant was excluded according to this criterion.

##### Test phase

During the Test phase, the participants performed the task in turn-taking with the virtual partner. The Test phase of each JA/Non-Int session comprised 288 trials, divided into 36 mini-blocks of 8 trials each. The session was divided into three blocks (12 mini-blocks each), divided by two breaks which had a variable duration that depended on the participant’s preference (the instruction was to press the key-button when ready to go on). Within each mini-block, each of the four melodies (JA task) or pairs of notes (Non-Int task) was presented once, in randomized order. We remind here that each musical sequence (melody/pair of notes) included two trials, where each trial consisted of the partner’s action plus the participant’s response. Instructions led participants to perform a G/C note 50% of times, and the physical congruence between the participant' and partner's actions (index-finger keypress action vs. middle-finger keypress action) was also randomized (50% of congruent/incongruent trials). Moreover, in each mini-block, in the 50% of the melodies/pairs of notes the action-note association was reversed in the partner (Reversed Association condition), while it never changed for the participants. In the Coherent Association trials, the participants interacted with a partner whose mouse worked identically to the participant’s one (i.e., pressing the index-finger button generates the C note, while pressing the middle-finger button generates the G note). Instead, in Reversed Association trials, the participants interacted with a partner whose mouse worked oppositely (i.e., pressing the index-finger button generates the G note, while pressing the middle-finger button generates the C note). Overall, participants performed 72 trials per each of the eight experimental conditions derived from the full-factorial combination of the following factors: Task (JA/Non-Int) × Association (Coherent/Reversed) × Congruency (Congruent/Incongruent action and space).

Importantly, in the JA condition the color cues indicate to the participants which notes both they and their partner have to play, thus creating expectations regarding the partner’s performance. This allowed us to create a further manipulation within the Reversed Association condition, and generate two types of “error” in the partner: (1) a Goal-Error, in which the partner presses the expected buttons but plays an unexpected note, and (2) an Action-Error, in which the partner performs an unexpected action (pressing the "wrong" button) but generates the expected note. 50% of the Reversed Association trials contained an Action-Error, and the remaining 50% a Goal-Error, leading to 36 trials per each Error-type. Moreover, 50% of each Error-type included a Matching versus a Not-Matching trial, i.e., a trial requiring a participant’s action that either matched or not with the action and note the participants expected to (but did not) see and hear from the partner (Fig. 2b). Trials were coded as following: each trial following a partner’s Action- or Goal-Error was coded according to the matching of the participant’s required action with the action that the partner was expected to (but did not) perform (Fig. 2b). Thus, a Matching trial is a trial where the participant’s response matches with a hypothetical correction of the partner’s error, while a Not-Matching trial requires the participant to do the opposite. For a better comprehension, we suggest that readers view the Supplementary Videos (Suppl. Videos 1–6) to gain a clear idea of the experimental manipulations.

The manipulations of Error-type and Matching-/Not-Matching-with-correction were not possible in the Non-Int condition as here the participants held no expectation of the partner's action. Yet, we tested whether participants in the Non-Int task realize that the partner’s action-note association is reversed in 50% of the trials. See Fig. 2a for a schematic representation of the whole experimental design.

Importantly, the Error-type and Matching-/Not-Matching-with-correction manipulations were controlled for perceptual Congruency, that is, the congruency between the partner’s actual action and note and those that the participant performed in the trial (see Table 3 below).

#### Trial timeline of the test phase

We counted as a “trial” each time participants performed a button press: thus, each musical sequence (the melodies in the JA condition and the pairs of notes in the Non-Int condition) consisted of two consecutive trials. Details on the trial timeline, which was identical in the JA and Non-Int conditions, are illustrated in Fig. 5.

At each trial, the partner always started first and then it was the participant’s turn. Each trial started with a fixation cross (500 ms), then the picture depicting the partner in the starting position was shown (200 ms); subsequently, the starting-position picture containing the color-cued instruction appeared (200 ms); next, the starting-position picture without the cue was shown again for 200 ms plus a variable stimulus onset asynchrony (soa) ranging from 100 to 700 ms; afterwards, the implied-motion picture was shown (100 ms) and followed by the final position picture, which was presented simultaneously with the partner’s note. The partner's note constituted the GO signal for the participants, who could then press one of the two mouse buttons: if the response was correct, the correct note would be played, otherwise, participants would hear the error signal. Participants were instructed to try and perform the task as quickly and correctly as possible.

Before starting each JA/Non-Int Test phase, a 16-trial familiarization block was provided.

### Data handling and design

We measured Accuracy (ACC), i.e., the proportion of correct responses, and Reaction Times (RTs), i.e., the time-delay between the go-signal and the participant’s button press measured in correct trials only.

Data were analyzed in the statistical programming environment R (R 3.3.3, R Core Team 2014). Bayesian analyses were implemented in JASP (Version 0.11.1., JASP Team, 2019 [Computer software]).

#### Analysis 1: preliminary analysis on the effect of Task (JA vs. Non-Int)

For the analysis of ACC, generalized linear mixed models for binomially distributed outcomes were used^86^. ACC data were submitted to a series of logistic mixed effect regressions using the GLMER procedure in the “lme4” R package^87^ (version 1.1-5). RTs were analyzed as a continuous dependent variable using linear mixed effects models, which were fitted using the LMER function in “lme4” R package^87^ (version 1.1-15); RTs values that fell 2.5 SDs above or below each individual's mean for each experimental condition were excluded from the analysis (434 trials in the whole sample, equal to the 3.37% of total correct trials). All participants showed an RTs grand mean that fell within 2.5 SDs above or below the group mean RTs. The group mean values in each experimental condition are reported for the sake of clarity in Table 1.

We considered as fixed effects the Task (JA vs. Non-Int), Association (Coherent vs. Reversed), Congruency (Congruent vs. Incongruent action and space), and their interactions. Concerning the random effect structure, by-subjects random intercepts were included to account for between-subject variability. The statistics of the fixed effects of the best fitting model were estimated with the “lmerTest” R package^88^ (version 3.0-1).

In the analysis of both ACC and RTs, the inclusion of a fixed effect in the final model was tested with a series of likelihood ratio tests (Suppl. Table S1 and S2). We only included the fixed effects that significantly increased the model’s goodness of fit^89^. The results of the best fitting models are reported in the main text. For the RTs, the significance levels are based on Satterthwaite’s degrees of freedom approximation. When appropriate, the post-hoc direct contrasts between the single levels of the significant interactions and main effects were conducted on the best fitting model with the “phia” R package^90^ (version 0.2-1) by applying Bonferroni correction for multiple comparisons. All tests of significance were based upon an α level of 0.05.

For the sake of clarity, we also report the same analysis performed on each Task separately. Here, we included the Association (Coherent vs. Reverse), Congruency (Congruent vs. Incongruent action and space), and their interactions, as fixed effects.

In Analysis1, we expected to find a Task × Association interaction indicating that a Reversed association impairs performance in the JA (as a result of an oPES-like effect) but not in the Non-Int condition.

#### Analysis 2: the effect of Error-type and correction tendencies in joint action

This analysis aimed to tested whether the observation-induced post-error slowing (oPES) that we expected to find in the JA task in Analysis1 was modulated by the Error-type (Action- vs. Goal-Error) and/or by the tendency to correct the partner’s mistake. We applied to the data collected during the JA task the analytical steps as described for the Analysis 1, based on generalized linear mixed models (used to analyze ACC) and linear mixed models (used to analyze RTs). We combined this with a Bayesian statistical approach that enabled us to measure the strength of evidence in favor of the null hypothesis when needed.

First, we considered as fixed effect only the Error-type (3 levels: trials following a partner’s action with no error vs. Action-Error vs. Goal-Error). In this analysis, a stronger oPES effect in trials following a partner’s Goal-Error as compared to Action-Error would be coherent with the Minimal Framework for joint action, while the absence of significant difference between the two Error-types would be in line with the DMP hypothesis.

Second, we considered as fixed effect the Matching with a hypothetical correction of the partner’s action (3 levels: trials following a partner’s action with no error versus trials where the participant’s action was Matching vs. Not-Matching with a hypothetical correction of the partner’s error, see Fig. 2b).

Finally, we checked whether, despite the absence of a significant difference between performance following a partner’s Action- or Goal-Error, this latter factor might nevertheless modulate the effect of Matching. As the Matching and Error-type factors were not orthogonal, because they both included the level “”no error”, before running an ANOVA on the RTs and the Inverse Efficiency Scores (IES^37^, i.e., the RTs/ACC ratio) data, we normalized the data collected in trials following a partner’s error by dividing them by the individuals’ performance in the trials following a partner’s correct action. This ANOVA included two within-subject factors, Error-type (Action- vs. Goal-Error) and Matching (Matching- vs. Not-Matching-with-correction conditions).

As the absence of significant difference between experimental conditions (e.g., between the Goal- vs.- Action-Error conditions) was one of the expected outcomes, we also planned to apply a Bayesian statistical analysis to assess the strength of evidence in favor of the null hypothesis in such instances. The rationale of this analysis is to consider the Bayes Factor (BF10) a statistical metric that quantifies the strength of evidence that the data provide in favor of the alternative hypothesis relative to the null hypothesis: a BF10 higher than 3 indicates substantial evidence in favor of the alternative hypothesis, whereas a BF10 lower than 0.3 indicates substantial evidence in favor of the null hypothesis^91^.

## Supplementary Information

Supplementary Information 1.

Supplementary Information 2.

Supplementary Information 3.

Supplementary Information 4.

Supplementary Information 5.

Supplementary Information 6.

Supplementary Information 7.

Supplementary Information 8.

## Data Availability

All relevant data are made available as Supplementary Dataset.
