# Supplementary material for: Mechanisms for mutual support in motor interactions
Source: Sci Rep. 2021 Feb 4;11:3060. doi: 10.1038/s41598-021-82138-y (PMC7862452; doi:10.1038/s41598-021-82138-y)
Supplement: Supplementary file 1 — Supplementary Information 1. [file 41598_2021_82138_MOESM1_ESM.docx]

**Mechanisms for mutual support in motor interactions**

Lucia Maria Sacheli^1,2^, Margherita Musco^1^, Elisa Zazzera^1^, Eraldo Paulesu^1,2^

1. Department of Psychology and Milan Center for Neuroscience (NeuroMi), University of Milano-Bicocca, Milan, Italy.

2. IRCCS Istituto Ortopedico Galeazzi, Milan, Italy.

Corresponding author:

Lucia Maria Sacheli

Department of Psychology, University of Milano-Bicocca,

Piazza dell'Ateneo Nuovo 1, 20126 Milano, Italy.

+39 02 64483776; [lucia.sacheli@unimib.it](mailto:lucia.sacheli@unimib.it)

**Supplementary Materials**

List of Supplementary Materials:

- Supplementary Tables S1 and S2 regarding the experiment described in the Main text.

- Supplementary Methods and Results regarding the Replication Experiment.

- Supplementary Videos 1-6.

- Supplementary Datafile containing all the relevant data on the Main and the Replication experiments.

## Supplementary Table S1. The results of the model comparison procedure performed in the Analysis1 on the Accuracy data of the experiment described in the Main text. The best fitting model is in bold.

| **Accuracy, all data** | | | | |
| --- | --- | --- | --- | --- |
| **Model** | **df** | **AIC** | **χ^2^** | ***p*** |
| ACC ~ 1 + (1 \| Participant) | 2 | 3203.7 |  |  |
| ACC ~ 1 + (1 \| Participant) + Task | 3 | 3084.9 | 120.78 | < .001 |
| ACC ~ 1 + (1 \| Participant) + Task * Association | 5 | 3062.2 | 26.74 | < .001 |
| **ACC ~ 1 + (1 \| Participant) + Task * Association * Congruency** | **9** | **3060.5** | **9.69** | **.046** |
| **Accuracy, Non-Int data only** | | | | |
| **Model** | **df** | **AIC** | **χ^2^** | ***p*** |
| ACC ~ 1 + (1 \| Participant) | 2 | 847.47 |  |  |
| **ACC ~ 1 + (1 \| Participant) + Congruency** | **3** | **844.78** | **4.70** | **.03** |
| ACC ~ 1 + (1 \| Participant) + Congruency * Association | 5 | 847.03 | 1.75 | .417 |
| **Accuracy, JA data only** | | | | |
| **Model** | **df** | **AIC** | **χ^2^** | ***p*** |
| ACC ~ 1 + (1 \| Participant) | 2 | 2233.8 |  |  |
| **ACC ~ 1 + (1 \| Participant) + Association** | **3** | **2209.0** | **26.80** | **< .001** |
| ACC ~ 1 + (1 \| Participant) + Association * Congruency | 5 | 2209.7 | 3.28 | .19 |

## Supplementary Table S2. The results of the model comparison procedure performed in the Analysis1 on the Reaction Times data of the experiment described in the Main text. The best fitting model is in bold.

| **Reaction Times, all data** | | | | |
| --- | --- | --- | --- | --- |
| **Model** | **df** | **AIC** | **χ^2^** | ***p*** |
| RT ~ 1 + (1 \| Participant) | 3 | 181955 |  |  |
| RT ~ 1 + (1 \| Participant) + Task | 4 | 180890 | 1067.78 | < .001 |
| **RT ~ 1 + (1 \| Participant) + Task * Association** | **6** | **180825** | **68.28** | **< .001** |
| RT ~ 1 + (1 \| Participant) + Task * Association * Congruency | 10 | 180825 | 8.19 | .084 |
| **Reaction Times, Non-Int data only** | | | | |
| **Model** | **df** | **AIC** | **χ^2^** | ***p*** |
| RT ~ 1 + (1 \| Participant) | 3 | 84557 |  |  |
| **RT ~ 1 + (1 \| Participant) + Congruency** | **4** | **84551** | **8.05** | **.004** |
| RT ~ 1 + (1 \| Participant) + Congruency * Association | 6 | 84552 | 3.60 | .166 |
| **Reaction Times, JA data only** | | | | |
| **Model** | **df** | **AIC** | **χ^2^** | ***p*** |
| RT ~ 1 + (1 \| Participant) | 3 | 91504 |  |  |
| **RT ~ 1 + (1 \| Participant) + Association** | **4** | **91458** | **47.30** | **< .001** |
| RT ~ 1 + (1 \| Participant) + Association * Congruency | 6 | 91460 | 2.54 | .280 |

**Supplementary Methods of the Replication Experiment.**

*Participants*. We report here the behavioral data of the 24 participants who took part in a functional MRI (fMRI) study (14 males, age range 28-19, m = 23.42 ± 1.91) in which the experimental procedure described in the Main Text was adapted to the fMRI environment. Participants were right-handed and reported normal or corrected-to-normal vision, absence of neurological or psychiatric disorders, and they were naive as to the purpose of the experiment. The protocol was approved by the ethics committee of the University of Milano-Bicocca (Italy) and was carried out according to the ethical standards of the 1964 Declaration of Helsinki and later amendments. Participants gave their written informed consent to take part in the study and were debriefed as to the purpose of the study at the end of the experimental procedures.

*Stimuli and Apparatus*. Stimuli were identical to the ones described in the Main Text. To adapt the paradigm to the timing required by fMRI data analysis, the implied-motion posture picture (depicting the pressing-button actions at mid-flight) was removed from the trial time-line. The experiment was performed inside a Siemens Magnetom Avanto 1.5 T scanner (Siemens AG, Erlangen, Germany). Visual stimuli were delivered though VisuaStim fiber-optic goggles (600x800 pixel resolution) and auditory stimuli via MRI-compatible headphones. The behavioral performance was collected through a standard device to record behavioral measures in the MRI scanner (Resonance Technology Inc., Northridge).

*Procedure.* Stimuli presentation and randomization were controlled by the E-Prime2 software (Psychology Software Tools Inc.). The experimental phases and conditions, and the number of trials, were identical to the ones described in the Main text. The trial time-line was also identical to the one described in the Main Text except for the absence of the implied-motion posture picture. The Learning phase was performed outside the scanner as described in the Main text, while the Test phase of the Non-Interactive and Joint Action tasks were performed during two different fMRI runs (counterbalanced order between participants).

*Data handling and design*. The behavioral data were analyzed as described in the Main text. Behaviorally, the aim of the replication experiment was to challenge the three crucial results that we found in the experiment described in the Main Text, that is: (i) the presence of a Task x Association significant interaction in Analysis1; (ii) the evidence in favor of the null hypothesis with regard to the difference between performance collected in Action-Error vs. Goal-Error trials (as tested by a Bayesian paired-sample t-test); and (ii) the presence of a main effect of Matching, and the absence of an interaction effect between Matching-with-correction and Error-type, as measured on the normalized RTs and Inverse Efficiency Scores data.

**Results.**

Replication of the Task x Association interaction (Analysis 1).

For the sake of clarity, we report in the table below (Supplementary Table S3) the group mean ACC and RT raw data: here, we calculated the individual mean ACC and RTs per each experimental condition by excluding from the calculation of RTs the outlier values that fell 2.5 SDs above or below each individual's mean for each experimental condition (accurate trials only).

**Supplementary Table S3**. Group mean ACC and mean RTs values in the Analysis 1 of the data collected during the Replication Experiment.

| **Accuracy** | | | | | | | | |
| --- | --- | --- | --- | --- | --- | --- | --- | --- |
|  | **JA-Coh-C** | **JA-Coh-In** | **JA-Rev-C** | **JA-Rev-In** | **NI-Coh-C** | **NI-Coh-In** | **NI-Rev-C** | **NI-Rev-In** |
| ***Mean*** | 0.98 | 0.97 | 0.96 | 0.95 | 0.98 | 0.98 | 0.98 | 0.98 |
| ***Range*** | 1.00-0.92 | 1.00-0.92 | 1.00-0.90 | 1.00-0.79 | 1.00-0.90 | 1.00-0.90 | 1.00-0.89 | 1.00-0.93 |
| **Reaction Times (ms)** | | | | | | | | |
|  | **JA-Coh-C** | **JA-Coh-In** | **JA-Rev-C** | **JA-Rev-In** | **NI-Coh-C** | **NI-Coh-In** | **NI-Rev-C** | **NI-Rev-In** |
| ***Mean*** | 341.76 | 379.73 | 388.51 | 372.60 | 301.81 | 311.37 | 305.42 | 316.48 |
| ***SD*** | 142.64 | 175.44 | 171.14 | 165.47 | 89.92 | 97.10 | 102.85 | 108.84 |

JA = Joint Action task, NI = Non-Interactive task; Coh = Coherent association, Rev = Reversed association;
C = Congruent, In = Incongruent.

*Accuracy*. The model including Task, Association, and their interaction, as fixed effect showed a significant main effect of Association (Wald Z = -4.88, *p* < 0.001) while the main effect of Task was not significant (Wald Z = 1.06, *p* = .29). The main effect of Association indicated that participants were more accurate in the trials in which the partner's action-note association was Coherent than Reversed (Coherent Association adj mean 0.983, SE 0.16, vs. Reversed Association adj mean 0.974, SE 0.15). Importantly, the results replicated the Task x Association significant interaction (Wald Z = 2.14, *p* = 0.032) indicating that the effect of Association was significant only in the JA task (Coherent Association adj mean 0.982, SE 0.18, vs. Reversed Association adj mean 0.964, SE 0.16, *p_corr_* < .001) and not in the Non-Int task (Coherent Association adj mean 0.985, SE 0.19, vs. Reversed Association adj mean 0.981, SE 0.18, *p_corr_* > .9).

*Reaction Times*. The model including Task, Association, and their interaction, as fixed effect showed a significant main effect of Task (F(1, 12899) = 522.38, *p* < 0.001) and Association (F(1, 12899) = 14.43, *p* < .001), indicating that participants were faster in the Non-Int than the JA task (adj mean 304.96 ms, SE 24.71 ms, vs. 365.70 ms, SE 24.71 ms), and in trials in which the partner's action-note association was Coherent than Reversed (adj mean 330.29 ms, SE 24.71 ms, vs. 340.38 ms, SE 24.71 ms). Importantly, the results also replicated the Task x Association significant interaction (F(1, 12899) = 6.47, *p* = .011) showing that the effect of Association was significant only in the JA task (Coherent Association adj mean 357.28 ms, SE 24.78 ms, vs. Reversed Association adj mean 374.12 ms, SE 24.78 ms, *p_corr_* < 0.001) and not in the Non-Int task (Coherent Association adj mean 303.29 s, SE 24.78, vs. Reversed Association 306.63 s, SE 24.78, *p_corr_* > .9).

Replication of the evidence in favor of the null hypothesis with regard to a possible difference between performance in the Action-Error and the Goal-Error trials.

We tested the model including as fixed effect only the Error-type (3 levels: trials following a partner’s action with no error vs. Action-Error vs. Goal-Error) on Accuracy and Reaction Times data. Both analyses replicated the results found in the experiment described in the Main text.

*Accuracy.* With regard to Accuracy, the results showed that the participants were more accurate in the trials following a partner’s correct action (adj mean 0.982, SE 0.18) than in the trials following a partner’s error (both ps_corr_ < .001), but the two error conditions did not differ from each other (p_corr_ > .9; Action-Error adj mean 0.964, SE 0.19, Goal-Error adj mean 0.966, SE 0.19).

*Reaction Times.* With regard to the Reaction Times, the results showed that the participants were faster in trials following a partner’s correct action (adj mean 357.39 ms, SE 32.61 ms) than in the trials following a partner’s error (Action-Error adj mean 379.24 ms, SE 32.73 ms, p_corr_ < .001; Goal-Error adj mean 370.63 ms, SE 32.72 ms, p_corr_ = .016), but the two error conditions did not differ from each other (p = .355).

Given the absence of a significant difference between performance in the Action- as compared to the Goal-Error conditions, we then tested the strength of evidence in favor of the null hypothesis with a Bayesian paired-sample t-test performed on the individual mean ACC and RTs in the Action- vs. Goal-Error conditions. The Bayesian Factor (BF10) was lower than 1, indicating evidence in favor of the null hypothesis, in both ACC and RTs. With regard to Accuracy, the BF10 was equal to 0.216, indicating moderate evidence in favor of the null hypothesis (mean ACC Action-Error 0.957 ± 0.05; mean ACC Goal-Error 0.958 ± 0.06). With regard to the Reaction Times, the BF10 was equal to 0.556, indicating anecdotal evidence in favor of the null hypothesis (mean RTs Action-Error 395.31 ± 176.19 ms; mean RTs Goal-Error 378.63 ± 117.80 ms).

Replication of the effect of Matching- vs. Not-Matching-with-correction.

First, we tested the model including as fixed-effect only the Matching with a hypothetical correction of the partner’s error (3 levels: trials following a partner’s action with no error vs. trials where the participant’s action was Matching-with-correction vs. Not-Matching-with-correction) on Accuracy and Reaction Times data. Both analyses replicated the results found in the experiment described in the Main text. With regard to Accuracy, the results showed that the participants were more accurate in the trials following a partner’s correct action (adj mean 0.982, SE 0.19) than in Not-Matching-with-correction trials (adj mean 0.955, SE 18, p_corr_ < .001), but not as comparted to Matching-with-correction trials (adj mean 0.974, SE 20, p_corr_ = .124). Accuracy in the Matching-with-correction trials was indeed higher than in Not-Matching-with-correction ones (p_corr_ = .002). With regard to the Reaction Times, the results showed that the participants were faster in trials following a partner’s correct action (adj mean 357.39 ms, SE 32.62 ms) than in the Not-Matching-with-correction trials (adj mean 393.78 ms, SE 32.74 ms, p < .001), while RTs did not show significant differences between trials following a partner’s correct action and Matching-with-correction trials (adj mean 356.62 ms, SE 32.74 ms, p > .9). Moreover, RTs in the Matching-with-correction trials were faster than in the Not-Matching-with-correction trials (p_corr_ < .001).

Then, we checked whether we could replicate the absence of a significant interaction between the effect of Matching-with-correction and Error-type. As in the experiment described in the Main text, this analysis was performed after having normalized each participant’s performance in each experimental condition following a partner’s error (i.e., Matching-with-correction in Action-Error, Matching-with-correction in Goal-Error, Not-Matching-with-correction in Action-Error, Not-Matching-with-correction in Goal-Error) by dividing it by the participant’s performance in trials following a partner’s correct action. We could thus run an ANOVA having Matching (2 levels: Matching- and Not-Matching-with-correction) and Error-type (2 levels: Action- and Goal-Error) as within-subject factors.

The results showed a significant effect of Matching for both RTs (F(1,23) = 7.16, p = .014, _p_η^2^ = .24) and IESs (F(1,23) = 10.61, p = .003, _p_η^2^ = .32), and the absence of a significant effect of Error-type and of a significant Matching x Error-type interaction (RT, all ps > .10; IES, all ps > .31).

**Supplementary Videos legends.**

**Suppl Video 1.** The video illustrates an example of two consecutive trials in the Joint Action task, which here form a melody in the Coherent Association condition. With regard to the explanatory voice, a standard voice taken from the Apple VoiceOver function has been used.

**Suppl Video 2.** The video illustrates an example of two consecutive trials in the Joint Action task, which here form a melody in the Coherent Association condition. With regard to the explanatory voice, a standard voice taken from the Apple VoiceOver function has been used.

**Suppl Video 3.** The video illustrates an example of two consecutive trials in the Joint Action task, which here form a melody in the Reversed Association condition. In this example, the trials contain a Goal-Error made by the partner, and the participant’s required responses match with a hypothetical correction of the partner’s error. With regard to the explanatory voice, a standard voice taken from the Apple VoiceOver function has been used.

**Suppl Video 4.** The video illustrates an example of two consecutive trials in the Joint Action task, which here form a melody in the Reversed Association condition. In this example, the trials contain a Goal-Error made by the partner, and the participant’s required responses do not match with a hypothetical correction of the partner’s error. With regard to the explanatory voice, a standard voice taken from the Apple VoiceOver function has been used.

**Suppl Video 5.** The video illustrates an example of two consecutive trials in the Joint Action task, which here form a melody in the Reversed Association condition. In this example, the trials contain an Action-Error made by the partner, and the participant’s required responses match with a hypothetical correction of the partner’s error. With regard to the explanatory voice, a standard voice taken from the Apple VoiceOver function has been used.

**Suppl Video 6.** The video illustrates an example of two consecutive trials in the Joint Action task, which here form a melody in the Reversed Association condition. In this example, the trials contain an Action-Error made by the partner, and the participant’s required responses do not match with a hypothetical correction of the partner’s error. With regard to the explanatory voice, a standard voice taken from the Apple VoiceOver function has been used.
